# National Outcomes of Venoarterial Extracorporeal Life Support in Patients with Chronic Kidney Disease

**DOI:** 10.1016/j.sopen.2025.04.011

**Published:** 2025-05-10

**Authors:** Oh Jin Kwon, Esteban Aguayo, Kevin Tabibian, Jeffrey Balian, Arjun Chaturvedi, Dariush Yalzadeh, Joseph Hadaya, Yas Sanaiha, Peyman Benharash

**Affiliations:** a Cardiovascular Outcomes Research Laboratories (CORELAB), David Geffen School of Medicine at University of California, Los Angeles, CA, United States of America; b Center for Advanced Surgical and Interventional Technology, Department of Surgery, David Geffen School of Medicine at University of California, Los Angeles, CA, United States of America; c Department of Surgery, David Geffen School of Medicine at University of California, Los Angeles, CA, United States of America; d Division of Cardiac Surgery, Department of Surgery, David Geffen School of Medicine at University of California, Los Angeles, CA, United States of America

## Abstract

**Background:**

Despite the increasing use of venoarterial extracorporeal membrane oxygenation (VA-ECMO) as advanced circulatory support for acute cardiac and circulatory failure, its high morbidity and mortality have necessitated the identification of risk factors. The prevalence of chronic kidney disease (CKD) in VA-ECMO patients remains unclear, and its relationship with outcomes is not well established.

**Methods:**

A retrospective analysis was conducted on patients (≥18 years) undergoing VA-ECMO using the 2019–2021 Nationwide Readmissions Database. Patients were stratified into *non-CKD*, *CKD 1–2*, and *CKD 3–5* based on renal disease severity. Those with end-stage renal disease requiring dialysis or prior renal transplant were excluded. The primary outcome was in-hospital mortality, while perioperative complications were secondarily assessed. Multivariable regression models were employed to assess the associations between CKD severity and outcomes across VA-ECMO indications.

**Results:**

Of an estimated 15,432 included for analysis, 11.7 % had CKD, with 84.7 % categorized as *CKD 3–5*. Following risk adjustment, *CKD 3–5* was independently associated with increased odds of in-hospital mortality (AOR 1.32, 95%CI 1.10–1.59) and overall complications (AOR 1.72, 95%CI 1.09–2.72) compared to *non-CKD*. Additionally, both *CKD 1–2* and *CKD 3–5* were linked to increased risks of cardiac and acute renal failure complications. When assessed across VA-ECMO indications, *CKD 3–5* was associated with the highest risk-adjusted mortality when used for postcardiotomy shock, cardiogenic shock, and mixed cardiopulmonary support.

**Conclusions:**

Advanced CKD is independently associated with increased mortality and perioperative complications in VA-ECMO patients, highlighting the association between preexisting renal dysfunction and adverse outcomes.

## Graphical abstract

**Figure:**
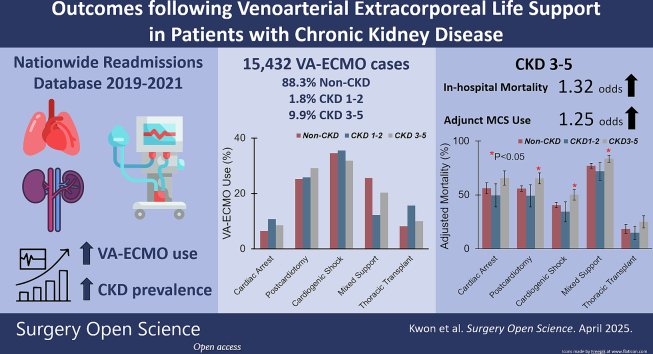

## Introduction

Venoarterial extracorporeal membrane oxygenation (VA-ECMO) serves as a life-sustaining intervention for patients with cardiogenic shock and cardiac arrest refractory to conventional therapies [1,2]. While extracorporeal membrane oxygenation (ECMO) provides temporary circulatory support, its benefit remains highly variable and depends on timely initiation and appropriate patient selection, as delayed deployment in patients with preexisting organ failure offers little survival advantage [[3], [4], [5], [6], [7], [8], [9]]. In emergent settings, clinical assessment is often limited, and ECMO may be initiated in critically ill patients in extremis with few remaining options, even when the likelihood of recovery is uncertain. Despite advancements in technology and the adoption of multidisciplinary shock teams to optimize decision-making, the absence of randomized trials and standardized candidacy criteria perpetuates uncertainty, particularly in high-risk populations with significant comorbidities or preexisting organ dysfunction.

Chronic kidney disease (CKD), a well-established predictor of adverse outcomes in critically ill patients, is recognized by the Extracorporeal Life Support Organization (ELSO) as a risk factor for increased mortality, prolonged mechanical ventilation, and extended ICU stays among ECMO recipients [[10], [11], [12], [13], [14], [15], [16]]. However, the extent to which CKD severity independently influences survival and complications remains poorly characterized. Existing literature and guidelines often aggregate CKD into composite risk models without stratifying outcomes by disease severity [[17], [18], [19], [20], [21]]. Given the hemodynamic and inflammatory stressors of VA-ECMO, patients with advanced CKD may be at heightened risk for secondary organ dysfunction, though these risks have not been clearly elucidated [[11], [12], [13], [14], [15], [16]]. In the urgent context of VA-ECMO initiation, improved characterization of the association between CKD severity and associated outcomes can refine patient selection and optimize perioperative management.

With the increasing prevalence of CKD and the widespread adoption of VA-ECMO utilization, further investigation into their relationship is warranted. In the present study, we utilized a contemporary, nationally representative cohort to examine the association of preexisting CKD across multiple severity stages on clinical outcomes, including in-hospital mortality and perioperative complications, among VA-ECMO recipients.

## Methods

This was a retrospective cohort study of the 2019–2021 Nationwide Readmissions Database (NRD). Maintained by the Agency for Healthcare Research and Quality as part of the Healthcare Cost and Utilization Project, the NRD is the largest all-payer readmissions database and uses survey weights to provide accurate estimates for approximately 60 % of US hospitalizations annually [22]. Using unique patient identifiers, the NRD tracks readmissions across hospitals within each calendar year. Due to the deidentified nature of the NRD, the present study was deemed exempt from full review by the Institutional Review Board at the University of California, Los Angeles (IRB 17–001112).

**Fig. 1:**
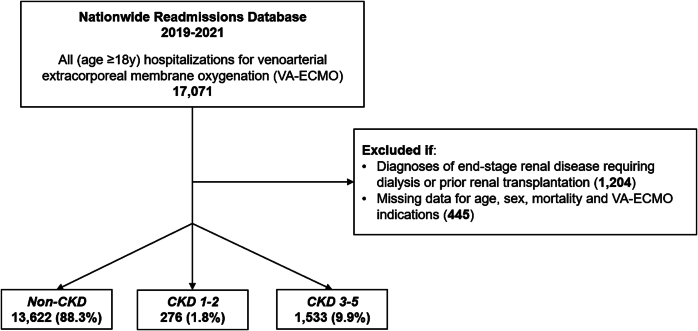
Consolidated Standards of Reporting Trials (CONSORT) diagram demonstrating study design. Patients were grouped into those without CKD (*non-CKD*), CKD stages 1–2 (*CKD 1–2*), and CKD stages 3–5 (*CKD 3–5*). CKD, chronic kidney disease.

All adult (≥18 years) hospitalizations for CKD patients receiving VA-ECMO were identified using previously reported International Classification of Diseases, 10th Revision (ICD-10) diagnosis and procedure codes [5,13,14,23]. Those with diagnoses of end-stage renal disease requiring dialysis or prior renal transplantation were not included to ensure a focus on patients with CKD not requiring renal replacement therapy. To account for center expertise, we separately tabulated the annual institutional volume of ECMO. Records missing data for age, sex, mortality, or VA-ECMO indications were also not considered (2.6 %) (Fig. 1).

Patient demographics and characteristics are reported as defined by the NRD data dictionary [22]. The Comorbid Operative Risk Evaluation (CORE) score was used to quantify the burden of comorbidities [24]. As detailed elsewhere, the CORE score is derived from captured comorbidities and provides a calibrated risk of mortality using machine learning algorithms. Furthermore, individual comorbidities corresponding to factors in the Society of Thoracic Surgeons Predicted Risk of Mortality Score were identified [25]. Patients were stratified based on the presence and severity of chronic renal dysfunction: without CKD (*non-CKD*), CKD stages 1–2 (*CKD 1–2*), and CKD stages 3–5 (*CKD 3–5*), as defined in previous literature [26]. To minimize misclassification of acute renal injury, ICD-10 diagnosis codes for acute renal failure were not included in defining CKD. Indications for VA-ECMO were defined using ICD-10 codes and categorized into cardiac arrest, postcardiotomy syndrome, and cardiogenic shock, following ELSO guidelines and prior literature [3,16,27]. Additional classifications included non-cardiogenic shock, representing VA-ECMO utilization for mixed cardiopulmonary support, and thoracic transplantation, which included patients undergoing heart or lung transplantation during the same index hospitalization.

The primary outcome was in-hospital mortality. Secondary outcomes included both individual and overall complications (neurologic, cardiac, respiratory, acute renal failure, infectious, hemorrhagic, thromboembolic, and acute limb ischemic events), postoperative length of stay (LOS), and unplanned readmissions at 30 and 90 days. We additionally assessed the use of other mechanical circulatory support (MCS) modalities, including intra-aortic balloon pump (IABP) and Impella devices.

Categorical data are reported as proportions and continuous data as means with standard deviations (SD) if normally distributed or as medians with interquartile range (IQR). Pearson's χ2, Mann–Whitney U, or adjusted Wald tests were used to assess the significance of intergroup differences, as appropriate. Multivariable logistic regression models were constructed to evaluate independent associations between CKD status and outcomes of interest. To evaluate the independent association between CKD severity and VA-ECMO outcomes, multivariable logistic regression models were constructed. Covariate selection was guided by the least absolute shrinkage and selection operator (LASSO) method to enhance model parsimony, reduce collinearity, and mitigate overfitting while prioritizing clinically relevant predictors. LASSO regression was applied as a variable selection tool rather than as a predictive modeling approach, ensuring that included covariates remained robust across multiple resampling iterations [28]. Models were selected to minimize the mean squared error term and evaluated using the receiver-operating characteristics curve and Akaike and Bayesian information criteria, as appropriate. Additional analyses were conducted to compare the distribution of VA-ECMO indications and associated outcomes across various groups of CKD. Adjusted outcomes of logistic regression analysis are reported as adjusted odds (AOR) with 95 % confidence intervals (CI). Statistical significance was set at α of 0.05. All statistical analyses were conducted using Stata 16.1 (StataCorp, College Station, TX).

## Results

Of an estimated 15,432 patients who underwent VA-ECMO, 11.7 % were identified as having a diagnosis of CKD, with the majority (84.7 %) classified as *CKD 3–5* and 15.3 % as *CKD 1–2*. Compared to the *non-CKD* cohort, patients with CKD were older (64 [55–71] vs 58 [45–66] years) and less frequently female (25.8 vs 35.4 %, both *P* < 0.001). Those in the *CKD 1–2* and *CKD 3–5* groups exhibited a higher prevalence of comorbidities, including chronic heart failure, coronary artery disease, and peripheral vascular compared to the *non-CKD* cohort (all P < 0.001, Table 1).

**Table 1:** Baseline characteristics of patients undergoing venoarterial extracorporeal membrane oxygenation (VA-ECMO), stratified by different stages of chronic kidney disease (CKD). Patients were grouped into without CKD (*non-CKD*), CKD stages 1–2 (*CKD 1–2*), and CKD stages 3–5 (*CKD 3–5*).

	*Non-CKD* (*n* = 13,623)	*CKD 1–2* (*n* = 276)	*CKD 3–5* (*n* = 1533)	*P*-value
Age (years)	58 [45–66]	60 [47–67]	64 [56–72]	**<0.001**
Female sex (%)	35.4	24.3	25.9	**<0.001**
CORE score	84 [67–94]	83 [65–92]	84 [67–93]	0.75
Comorbidities (%)				
Cerebrovascular disease	4.3	8.5	7.8	**<0.001**
Coronary artery disease	54.6	61.0	67.7	**<0.001**
Chronic heart failure	7.5	12.6	16.1	**<0.001**
Cardiomyopathy	20.4	31.0	31.2	**<0.001**
Valvular heart disease	14.3	17.9	20.9	**<0.001**
Cardiac arrhythmias	66.8	67.8	76.0	**<0.001**
Hypertension	55.7	86.2	90.0	**<0.001**
Chronic pulmonary disease	17.2	26.2	18.1	**0.04**
Pulmonary vascular disease	16.9	26.2	27.7	**<0.001**
Peripheral vascular disease	17.7	31.7	30.6	**<0.001**
Chronic liver disease	41.7	31.7	40.5	0.08
Coagulopathy	57.6	58.5	63.6	**0.02**
Diabetes mellitus	26.0	44.9	47.4	**<0.001**
Hyperlipidemia	11.1	14.3	14.9	**0.02**
Anticoagulants or antiplatelets use	10.1	21.0	16.8	**<0.001**
Prior pacemaker or ICD	3.8	13.5	13.5	**<0.001**
Smoking history	26.2	38.3	24.1	**0.01**
Admission to VA-ECMO interval (days)	1 [0–4]	2 [0–7]	3 [1–8]	**<0.001**

CORE, Comorbid Operative Risk Evaluation; ICD, implantable cardioverter-defibrillator.

Categorical data are expressed as % and continuous data are expressed as median [interquartile range]. Bold type denotes P-value <0.05.

**Fig. 2:**
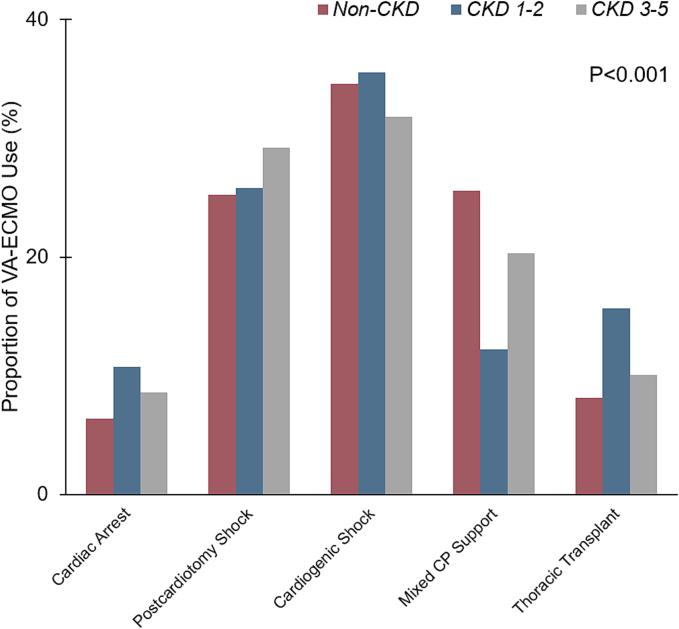
Proportion of venoarterial extracorporeal membrane oxygenation (VA-ECMO) use, stratified by operative indications and severity of chronic kidney disease (CKD). Patients were grouped into without CKD (*non-CKD*), CKD stages 1–2 (*CKD 1–2*), and CKD stages 3–5 (*CKD 3–5*). Thoracic transplantation was defined as heart or lung transplantation performed after undergoing VA-ECMO during the index hospitalization. CP, cardiopulmonary.

The proportion of VA-ECMO utilization across clinical indications, stratified by different stages of CKD, is illustrated in Fig. 2. When assessed across different indications, significant variations in the distribution of VA-ECMO use were noted (*P* < 0.001), with cardiogenic shock accounting for the highest proportion (34.3 %) across all groups. Moreover, patients with renal dysfunction required longer intervals from admission to VA-ECMO initiation compared to those without CKD, with median times of 1 [0–4], 2 [0–7], and 3 [1–8] days for *non-CKD*, *CKD 1–2*, and *CKD 3–5* groups, respectively (*P* < 0.001, Table 1).

On unadjusted analysis, in-hospital mortality was highest among patients in the *CKD 3–5* group (60.2 %), followed by the *non-CKD* (53.1 %) and *CKD 1–2* groups (41.7 %) (P < 0.001, Table 2). Although overall complication rates were comparable across these groups (*P* = 0.09), significant differences in individual complications were noted. Those with preexisting renal dysfunction more frequently experienced cardiac and acute renal failure complications and required adjunctive MCS during the perioperative period (Table 2).

**Table 2:** Unadjusted outcomes of patients undergoing venoarterial extracorporeal membrane oxygenation (VA-ECMO), stratified by different stages of chronic kidney disease (CKD). Patients were grouped into without CKD (*non-CKD*), CKD stages 1–2 (*CKD 1–2*), and CKD stages 3–5 (*CKD 3–5*).

	*Non-CKD* (n = 13,623)	*CKD 1–2* (n = 276)	*CKD 3–5* (n = 1533)	P-value
In-hospital mortality (%)	53.1	41.7	60.2	**<0.001**
Overall complications (%)	95.0	96.5	96.7	0.09
Individual complications (%)				
Neurologic	14.2	7.5	13.4	0.11
Cardiac	64.8	74.7	76.7	**<0.001**
Respiratory	69.3	59.2	65.8	**0.01**
Acute limb ischemia	8.8	5.6	6.0	**0.01**
Acute renal failure	74.4	81.9	88.0	**<0.001**
Infectious	31.9	19.1	29.0	**0.01**
Hemorrhagic	71.1	67.2	75.4	**0.03**
Thromboembolic	23.2	21.8	14.0	**<0.001**
Adjunctive mechanical circulatory support	39.3	41.2	50.6	**<0.001**
Postoperative length of stay (days)	22 [11–37]	20 [13−33]	23 [10–41]	0.31
Non-elective 30-day readmission (%)	14.3	19.3	18.2	0.12
Non-elective 90-day readmission (%)	15.8	25.0	20.2	**0.01**

Categorical data are expressed as % and continuous data are expressed as median [interquartile range]. Bold type denotes P-value <0.05.

**Fig. 3:**
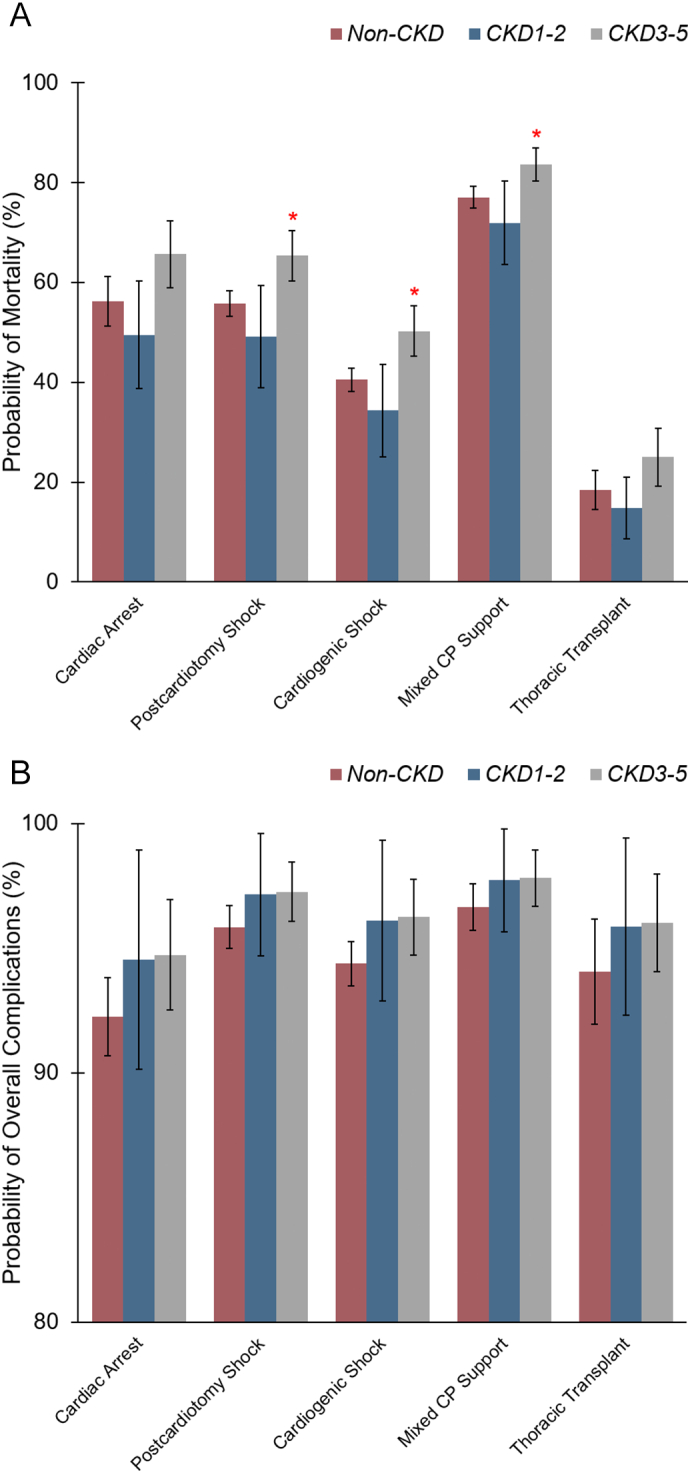
Risk-adjusted probability of (A) in-hospital mortality and (B) overall complications following venoarterial extracorporeal membrane oxygenation (VA-ECMO), stratified by operative indications and the severity of chronic kidney disease (CKD). Patients were grouped into without CKD (*non-CKD*), CKD stages 1–2 (*CKD 1–2*), and CKD stages 3–5 (*CKD 3–5*). Thoracic transplantation was defined as heart or lung transplantation performed after undergoing VA-ECMO during the index hospitalization. CP, cardiopulmonary. *P < 0.05.

Following multivariable adjustment, *CKD 3–5* was independently associated with increased odds of in-hospital mortality (AOR 1.32, 95 % CI 1.10–1.59) and overall complications (AOR 1.72, 95 % CI 1.09–2.72) relative to the *non-CKD* group. Specifically, both *CKD 1–2* and *CKD 3–5* remained linked to higher risks of cardiac and acute renal failure compared to their *non-CKD* counterpart (Table 3). However, only *CKD 3–5* was shown to increase the risk for adjunctive MCS use (AOR 1.25 95 % CI [1.05–3.68]). Analysis stratified by operative indications demonstrated that *CKD 3–5* was associated with the highest risk-adjusted rate of mortality, particularly in patients undergoing VA-ECMO for postcardiotomy and cardiogenic shocks, as well as mixed cardiopulmonary support (Fig. 3A).

**Table 3:** Risk-adjusted outcomes following venoarterial extracorporeal membrane oxygenation (VA-ECMO), stratified by different stages of chronic kidney disease (CKD), with those without CKD as the reference group. Patients were grouped into without CKD (*non-CKD*), CKD stages 1–2 (*CKD 1–2*), and CKD stages 3–5 (*CKD 3–5*).

	*CKD 1**–**2*		*CKD 3**–**5*	
(Reference: *non-CKD*)	AOR [95 % CI]	*P*-value	AOR [95 % CI]	P-value
In-hospital mortality	0.80 [0.52–1.23]	0.31	1.32 [1.10–1.59]	**0.01**
Overall complications	1.95 [0.74–5.10]	0.18	1.72 [1.09–2.72]	**0.02**
Individual complications				
Neurologic	0.53 [0.28–1.01]	0.05	0.90 [0.68–1.19]	0.45
Cardiac	1.98 [1.16–3.36]	**0.01**	1.58 [1.22–2.06]	**0.001**
Respiratory	0.68 [0.44–1.07]	0.10	0.89 [0.73–1.07]	0.22
Acute limb ischemia	0.68 [0.36–1.31]	0.25	0.75 [0.55–1.03]	0.07
Acute renal failure	2.10 [1.34–3.30]	**0.001**	2.85 [2.22–3.66]	**<0.001**
Infectious	0.63 [0.41–0.99]	**0.04**	1.01 [0.84–1.20]	0.95
Hemorrhagic	0.85 [0.59–1.22]	0.39	1.12 [0.93–1.33]	0.23
Thromboembolic	1.07 [0.65–1.76]	0.80	0.69 [0.52–0.90]	**0.01**
Adjunctive mechanical circulatory support	1.02 [0.70–1.49]	0.92	1.25 [1.05–3.66]	**0.01**
Non-elective 30-day readmission	1.35 [0.78–2.32]	0.28	1.25 [0.86–1.81]	0.24
Non-elective 90-day readmission	1.86 [1.16–2.97]	**0.01**	1.32 [0.97–1.79]	0.08

Mechanical circulatory support was defined as perioperative intra-aortic balloon pump or Impella use.

AOR, adjusted odds ratio; CI, confidence intervals.

Bold type denotes P- value <0.05.

## Discussion

To our knowledge, this is the first nationally representative study to examine the association between preexisting CKD severity and VA-ECMO outcomes. Our findings demonstrate that advanced stages of CKD are independently associated with an increased risk of in-hospital mortality and perioperative complications. Furthermore, specific complications, such as cardiac events and acute renal failure, remained higher in the presence of CKD, irrespective of severity. While the distribution of clinical indications for VA-ECMO initiation was similar across CKD stages, advanced CKD demonstrated higher risk-adjusted mortality in specific operative contexts. Several of these findings have significant implications for understanding the interplay between CKD and VA-ECMO and warrant further discussion.

Significant morbidity and mortality associated with VA-ECMO have long driven efforts to refine patient selection and improve risk stratification. Over the past two decades, several predictive models such as the Survival After Venoarterial ECMO (SAVE), the Extra-Corporeal Membrane Oxygenation for Refractory Cardiogenic Shock (ENCOURAGE), and the Prediction of Early Mortality Associated with VA-ECMO Using Preimplantation Characteristics (IMPACT) scores have consistently identified chronic renal dysfunction as a key determinant of survival in this high-risk population [[17], [18], [19], [20], [21]]. However, a critical limitation of these models lies in their treatment of CKD as a binary variable (present or absent), obscuring the graded risk inherent to renal dysfunction. This oversimplification fails to account for the profound physiological differences between early and advanced CKD, distinctions that carry significant prognostic implications. In our nationally representative cohort, over 80 % of patients with CKD had advanced disease, a striking predominance of severe renal disease that underscores the clinical urgency of stage-specific risk assessment.

The omission of CKD staging in prior analyses has practical consequences. Advanced CKD reflects cumulative vascular calcification, chronic inflammation, and endothelial dysfunction – pathophysiological hallmarks that amplify susceptibility to ECMO-related complications such as ischemic injury, thromboembolism, and multiorgan failure [29,30]. These systemic derangements impair renal autoregulation, exacerbate fluid overload, and heighten oxidative stress, compounding the inherent risks of extracorporeal circulation. By contrast, early-stage CKD may retain sufficient renal reserve to buffer these insults. Our findings validate this distinction. Advanced CKD was associated with a 35 % higher risk of mortality and a 72 % increase in complications compared to non-CKD counterparts, whereas early-stage CKD did not demonstrate independent mortality or overall complications risks. This observed stage-dependent risk gradient calls into question the adequacy of binary CKD classification in existing VA-ECMO risk models. For instance, the 40 % increase in mortality risk associated with CKD in the widely adopted SAVE score may underestimate risk for advanced stages while overestimating for early disease [17]. Such imprecision risks overtreatment of lower-risk patients and undertreatment of those who might benefit from timely VA-ECMO despite advanced CKD. By quantifying this gradient, this study provides a framework to refine predictive tools and ensure risk stratification aligns with the biological continuum of renal dysfunction.

Beyond diminished physiological reserve, the extensive comorbidity profiles associated with CKD, including coronary artery disease, diabetes, and heart failure, complicate perioperative management and may delay VA-ECMO initiation. Notably, in the present analysis, advanced CKD patients experienced the longest delays to ECMO deployment. Although the lack of significant difference in CORE score across CKD strata is noteworthy, this may reflect its limitation in accounting for disease severity within individual domains. Assigning equivalent weights to early and advanced CKD likely obscures the physiological gradient of risk associated with progressive renal dysfunction and may diminish the utility of such indices in guiding timely risk stratification for VA-ECMO candidates. While inherent database limitations preclude definitive conclusions about the specific drivers of these delays, the observed differences raise reasonable speculation that prolonged risk stratification, comorbidity optimization, or multidisciplinary consultations may have contributed to high-risk cases. Previous studies have reported similar trends, with patients burdened by multiple comorbidities encountering prolonged delays to intervention [31,32]. Yet, emerging evidence suggests a survival benefit of earlier ECMO initiation, particularly within 48 h of shock onset, which mitigates hypoperfusion-related end-organ dysfunction [31]. While thorough evaluation remains essential, delays in VA-ECMO initiation risk irreversible multiorgan dysfunction, diminishing its benefits in salvageable patients. In advanced CKD, achieving the balance between timely VA-ECMO initiation and careful risk assessment is crucial, as prolonged preprocedural optimization may trade reversible shock for irreversible collapse. Future studies must define optimal timing thresholds and develop strategies that facilitate comprehensive evaluation while ensuring timely ECMO deployment in this high-risk population.

Efforts to understand the increased incidence of organ-specific injuries in those with preexisting renal dysfunction undergoing VA-ECMO highlight potential mechanistic contributors that extend beyond baseline kidney injury. While VA-ECMO may transiently improve overall perfusion, these benefits often fail to counteract diminished nephron reserve and microvascular compromise inherent to CKD, thereby predisposing kidneys to ischemic injury and acute renal failure [30,33]. Similarly, structural and functional cardiac alterations associated with CKD, coupled with the elevated left ventricular afterload imposed by the extracorporeal circuit, may explain the increased risk of cardiac complications in our analysis [[34], [35], [36]]. In many cases, a greater reliance on additional, adjunctive MCS in this group may reflect attempts to achieve hemodynamic stability amid these overlapping challenges. However, the potential risks surrounding adjunctive MCS utilization, including infection, vascular trauma, and bleeding, highlight the potential downsides and the need to balance their benefits with the hazards of escalating therapy [30]. Ultimately, targeted measures prioritizing organ protection, vigilant hemodynamic monitoring, and multidisciplinary management may hold promise for mitigating complications in this vulnerable population.

As current guidelines suggest, the decision to initiate extracorporeal support should be primarily guided by immediate clinical necessity [16]. Prior literature has similarly reported that the severity and reversibility of the primary indication for VA-ECMO often outweigh baseline comorbidities in determining survival [3,27]. In the present analysis, although the overall distribution of VA-ECMO indications was similar across varying degrees of renal impairment, patients with advanced CKD experienced disproportionately higher mortality when undergoing extracorporeal support for postcardiotomy shock, cardiogenic shock, or mixed cardiopulmonary conditions. Our findings may suggest that preexisting organ impairment might impose additional perioperative risks and limit the capacity of the extracorporeal circuit to compensate for severe postcardiotomy and cariogenic states. While comorbid conditions alone should not preclude the use of VA-ECMO when the underlying condition is potentially reversible, our findings support current guidelines advocating for an individualized approach that considers patient-specific factors to optimize selection criteria, timing, and perioperative management [16]. Future efforts should refine risk models to account for this interplay, ensuring ECMO deployment balances urgency with physiological reserve.

Findings of this study should be interpreted in the context of several limitations inherent to its retrospective and observational design. The use of an administrative database relying on ICD-10 coding limits the granularity of patient data, including precise measures of renal function such as glomerular filtration rate and albuminuria levels. Additionally, the database lacks detailed information on ECMO-related parameters, such as duration of VA-ECMO support, cannulation strategies, flow rates, and anticoagulation protocols, all of which could influence patient outcomes. Furthermore, unmeasured confounders such as institutional practices, surgeon expertise, and specific management protocols may have influenced the decision to initiate VA-ECMO and its timing, introducing potential selection bias. While we adjusted for observable patient- and hospital-level factors using multivariable modeling and LASSO-based covariate selection, residual confounding from unmeasured variables cannot be excluded. Therefore, adjusted estimates should be interpreted with appropriate caution. Despite these limitations, the use of a large, nationally representative database allows for the assessment of outcomes across broader populations and diverse practice settings. Prior studies have demonstrated the utility of such datasets in evaluating ECMO-related outcomes, resource utilization, and disparities in access to advanced therapies [3,4,27]. Importantly, subgroups such as patients with CKD, particularly when stratified by severity, remain underrepresented in prospective trials and ECMO registries, highlighting the value of nationally representative data in informing practice. Further prospective and randomized control trials, however, are warranted to validate these associations and inform treatment decisions.

In conclusion, the majority of VA-ECMO recipients with CKD had advanced-stage disease, which was independently associated with increased perioperative in-hospital mortality and perioperative complications. Moreover, preexisting CKD was linked to higher rates of renal and cardiac complications, as well as greater reliance on adjunctive MCS. Notably, mortality risk disparities persisted across clinical indications, with advanced CKD patients demonstrating disproportionately higher mortality in postcardiotomy shock, cardiogenic shock, and mixed cardiopulmonary support. Our findings suggest the need to integrate CKD severity into clinical decision-making frameworks to refine patient selection and optimize perioperative management in this high-risk population.

## CRediT authorship contribution statement

**Oh Jin Kwon:** Writing – review & editing, Writing – original draft, Validation, Methodology, Formal analysis, Data curation, Conceptualization. **Esteban Aguayo:** Writing – review & editing, Writing – original draft, Methodology. **Kevin Tabibian:** Writing – review & editing, Methodology, Formal analysis. **Jeffrey Balian:** Writing – review & editing, Methodology, Conceptualization. **Arjun Chaturvedi:** Formal analysis, Conceptualization. **Dariush Yalzadeh:** Writing – review & editing, Conceptualization. **Joseph Hadaya:** Writing – review & editing, Validation, Methodology. **Yas Sanaiha:** Writing – review & editing, Supervision, Conceptualization. **Peyman Benharash:** Writing – review & editing, Validation, Supervision, Methodology.

## Conflicts of interest/disclosures

Dr. Peyman Benharash received fees from AtriCure as a surgical proctor. This manuscript does not discuss any AtriCure products or services. The remaining coauthors have no related conflicts of interest to disclose. This research did not receive any funding from agencies in the public, commercial, or not-for-profit sectors.

## Ethics approval

Due to the deidentified nature of the Nationwide Readmissions Database (NRD), the present study was deemed exempt from full review by the Institutional Review Board at the University of California, Los Angeles (IRB 17–001112).

## Funding/financial support

The present study did not receive any funding or financial support from extramural sources including agencies in the public, commercial, or not-for-profit sectors.

## Declaration of competing interest

The authors declare that they have no known competing financial interests or personal relationships that could have appeared to influence the work reported in this paper.
